# Materials, Design, and Characteristics of Bulk Acoustic Wave Resonator: A Review

**DOI:** 10.3390/mi11070630

**Published:** 2020-06-28

**Authors:** Yan Liu, Yao Cai, Yi Zhang, Alexander Tovstopyat, Sheng Liu, Chengliang Sun

**Affiliations:** Institute of Technological Sciences, Wuhan University, Wuhan 430072, China; liuyan92@whu.edu.cn (Y.L.); caiyao999@whu.edu.cn (Y.C.); zhang.yi@whu.edu.cn (Y.Z.); alxtov@whu.edu.cn (A.T.); Victor_liu63@vip.126.com (S.L.)

**Keywords:** aluminum nitride, bulk acoustic wave resonator, frequency tuning, radio frequency (RF) integration, spurious mode, temperature compensation

## Abstract

With the rapid commercialization of fifth generation (5G) technology in the world, the market demand for radio frequency (RF) filters continues to grow. Acoustic wave technology has been attracting great attention as one of the effective solutions for achieving high-performance RF filter operations while offering low cost and small device size. Compared with surface acoustic wave (SAW) resonators, bulk acoustic wave (BAW) resonators have more potential in fabricating high- quality RF filters because of their lower insertion loss and better selectivity in the middle and high frequency bands above 2.5 GHz. Here, we provide a comprehensive review about BAW resonator researches, including materials, structure designs, and characteristics. The basic principles and details of recently proposed BAW resonators are carefully investigated. The materials of poly-crystalline aluminum nitride (AlN), single crystal AlN, doped AlN, and electrode are also analyzed and compared. Common approaches to enhance the performance of BAW resonators, suppression of spurious mode, low temperature sensitivity, and tuning ability are introduced with discussions and suggestions for further improvement. Finally, by looking into the challenges of high frequency, wide bandwidth, miniaturization, and high power level, we provide clues to specific materials, structure designs, and RF integration technologies for BAW resonators.

## 1. Introduction

The rapid development of wireless communication applications has led to the urgent requirement for wide bandwidth and high-speed data transmissions. In the field of mobile communications, the fifth generation (5G) technology is the new technology after 4G. Compared with 3G and 4G, the transmission rate and the ability to carry data of 5G networks have been greatly improved with a high utilization of spectrum resources and complex communication protocols. Nowadays, more than 30 bands are being used, and this number keeps increasing. In order to better satisfy the ever-increasing requirement, radio frequency (RF) filters have been developed dramatically.

**Figure 1:**
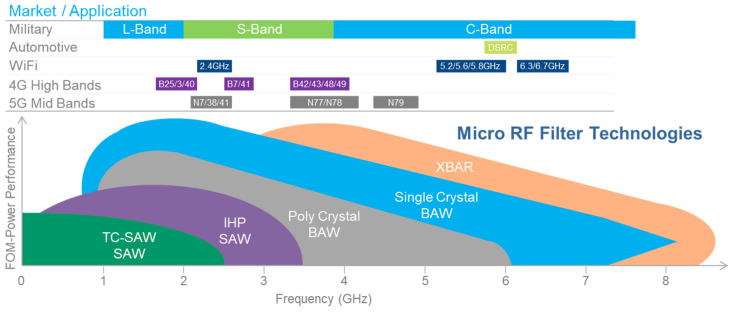
Market application and band allocation of radio frequency (RF) filter technologies [5].

At present, the most mainstream implementation of RF filters is dominated by surface acoustic wave (SAW) filters and bulk acoustic wave (BAW) filters. A milestone of micro RF filter technologies is presented in Figure 1. When compared with SAW filters, BAW filters are widely acknowledged because of their high operating frequency and power capacity advantages. The high-quality factor (Q-factor) of BAW makes filter skirts steeper, while high acoustic velocity and thermal conductivity enable high power handling. Additionally, the low power consumption, high isolation, and complementary metal oxide semiconductor (CMOS) compatibility of BAW filters make it a dominant device in the field of RF communications [1]. In 1999, the first commercially valuable film BAW duplexer was developed by the American company Agilent [2] and it was officially commercialized two years later [3,4]. Subsequently, lots of international well-known semiconductor companies, including Broadcom (Avago), Qorvo, Taiyo, Samsung, Infineon, STMicroelectronics, EPCOS and Fujitsu, have devoted a huge amount of effort to this area.

In many ways, the emergence for 5G is a challenge for data rates, coverage, and minimization. Firstly, the phases of 5G utilize higher radio frequencies, but the acoustic and ohmic losses increase dramatically with operating frequency of BAW filters. Secondly, the N78, N79, and N77 of 5G NR frequency bands require 500, 600, and 900 MHz of bandwidth, respectively, while obtaining a relatively large bandwidth, up to 10%, is also a difficult problem for a BAW filter based on aluminum nitride (AlN). Thirdly, considering the entire RF system, the decreasing module size and increasing input power are inevitable trends in the future. 

The BAW resonator, as a basic unit of RF circuits, significantly affects the performance of BAW filters. AlN is still the preferential material for bulk production of BAW devices, but the most limiting property is probably the coupling coefficient. In this work, we summarize the existing technologies of BAW resonators, and discuss merits of various piezoelectric and electrode materials in high-coupling, large-bandwidth, high-performance designs. Next, the common methods and latest designs for optimizing the spurious mode, temperature compensation, and multi-band of the resonator are given. Finally, a new design of laterally-excited bulk-wave resonators (XBAR) with ~25% strong piezoelectric coupling and a RF integration technology are proposed for further improving the properties of BAW devices, thus enhancing the availability of BAW resonators and filters in RF communication.

## 2. The Basic Principle

### 2.1. Structure of Resonator

**Figure 2:**
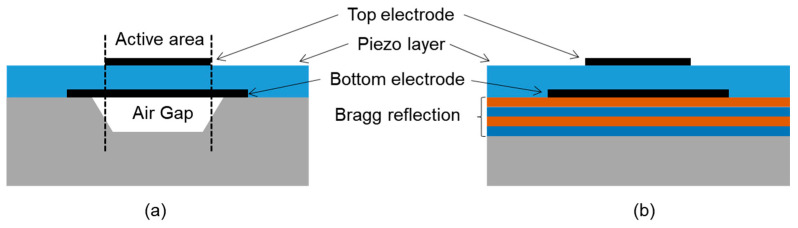
Structure of (**a**) the film bulk acoustic resonator (FBAR) formed over an air gap and (**b**) the solid mounted resonator (SMR) formed over a Bragg reflection layer.

The BAW resonator is a piezoelectric stack structure consisting of a piezoelectric film sandwiched between two metallic electrodes. The area where the upper and lower electrodes and the piezoelectric layer overlap in the thickness direction is defined as the active area of the resonator. The FBAR (film bulk acoustic resonator) and SMR (solid mounted resonator) are two types of technology of BAW resonators (Figure 2). The fundamental resonance occurs when the wavelength of the mechanical wave generated by the excitation is twice the thickness of the piezoelectric layers. Regardless of the electrodes, the resonant frequency depends on the acoustic velocity and the thickness of the piezoelectric film:

(1) $$f_{0}=\nu /2d$$

where *f*_0_ and *v* are the resonant frequency and acoustic velocity, respectively, and *d* is the thickness of the piezoelectric film. Also, the thickness of the electrode can affect the resonant frequency. An electrode of different thickness as a mass loading can generate a frequency shift, which is often used to form the filter passband. 

### 2.2. Equivalent Model

**Figure 3:**
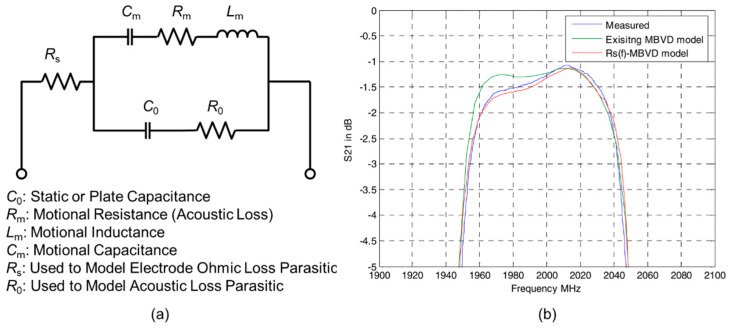
(**a**) Modified Butterworth Van Dyke (MBVD) model [8]. (**b**) Measured filter data of *R*_s_(*f*)-MBVD model, reproduced with permission from [9].

The physical one-dimensional Mason model and circuit-based modified Butterworth Van Dyke (MBVD) model are commonly used for BAW resonator and filter design. The input and output impedance characteristics of the resonator can be obtained directly using the Mason model with the equivalent mathematical deformation of the structure and material parameters, and the energy loss mechanism can be described by introducing a complex modulus of elasticity and a complex propagation constant [6]. The MBVD model is proposed to describe the electrical properties of BAW resonator near low frequency or resonance point (Figure 3a). The parasitic parameters *R*_s_ and *R*_0_ are introduced and used to illustrate the electrical loss of electrode and dielectric loss of piezoelectric film. Particularly, the parasitic parameters extracted by the MBVD model can be used in the Mason model for a better fit [7]. 

**Figure 4:**
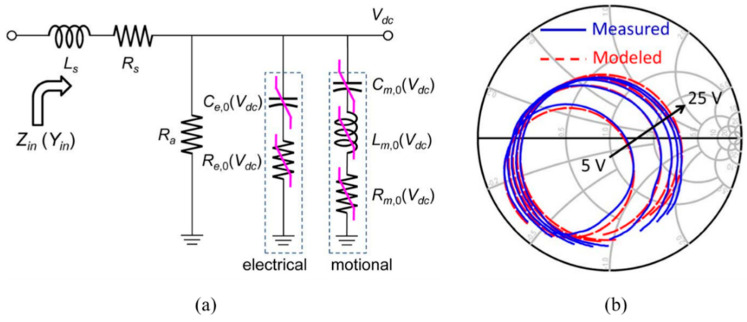
(**a**) Nonlinear MBVD model. (**b**) The measured and modeled reflection coefficients on the Smith chart with DC bias voltages. Reproduced with permission from [10].

Frank Z. Bi et al. proposed a model called *R*_s_(*f*)-MBVD [9] to solve the problem that the traditional MBVD model cannot fit the impedance of resonator due to spurious mode loss below *f*_s_. By replacing *R*_s_ with *R*_s_+*r*_s_(*f*), where *r*_s_(*f*) is the introduced frequency dependent resistance, the newly improved MBVD model can predict filter passband slope distortion for filter design and optimization (Figure 3b). Seungku Lee et al. [10] proposed a nonlinear MBVD model for switchable Ba*_x_*Sr_1-*x*_TiO_3_ (BST) ferroelectric FBARs. A method for extracting the voltage-dependent (*V*_dc_) MBVD model parameters (i.e., *L*_m,0_, *C*_m,0_, *R*_m,0_, *C*_e,0_, and *R*_e,0_) and a model applicable over a wide range of DC bias voltage is developed, as is shown in Figure 4. 

### 2.3. Key Parameters of Bulk Acoustic Wave (BAW) Resonator

For the BAW resonator, the figure of merit (FOM) is one of the most important parameters, and can be defined as:

(2) $$\mathrm{FOM}=k_{\mathrm{eff}}{}^{2}\times Q$$

where *k*_eff_^2^ is the effective coupling coefficient, and Q is the quality factor. A larger *k*_eff_^2^ gives a wide bandwidth, which is desired for 5G band. Increasing the resonator Q-value (especially such that both Q_s_ and Q_p_ values are greater than 2000) is a significant goal of resonator design.

#### 2.3.1. Effective Coupling Coefficient

Piezoelectric material-coupling coefficient *k*_t_^2^ and effective coupling coefficient *k*_eff_^2^ are two different expressions. It is not hard to design a resonator with the required *k*_eff_^2^ from a material having a quite adequate *k*_t_^2^ value [11]. Besides, electrode configuration, acoustic reflector, and parasitics are other major factors that can significantly influence the *k*_eff_^2^ [12,13]. The calculation of *k*_eff_^2^ via series resonant frequency *f*_s_ and parallel resonant frequency *f*_p_ is given [8]:

(3) $$k_{\mathrm{eff}}^{}{}^{2}=\frac{\pi^{2}}{4}\times \frac{f_{s}}{f_{p}}\times (\frac{f_{p}-f_{s}}{f_{p}})$$

**Figure 5:**
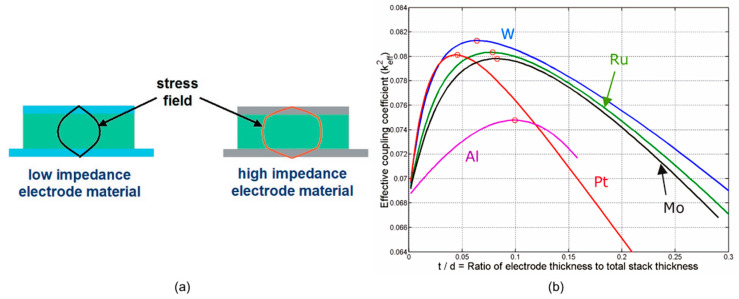
(**a**) Stress field distribution throughout a bulk acoustic wave (BAW) resonator with low impedance and high impedance electrode materials, reproduced with permission from [12]. (**b**) Relationship between *k*_eff_^2^ and the ratio of electrode thickness to total stack thickness for an AlN resonator with different electrode materials, reproduced with permission from [14].

When AlN is deposited on the substrate, a transition region with poor crystal quality will be formed at the interface between the AlN and the substrate. The thinner this layer is, the better the overall layer coupling will be [5]. For a given material, there is always an optimum thickness ratio that maximizes the *k*_eff_^2^ (see Figure 5b). As for electrodes, high acoustic impedance materials can boost the achievable *k*_eff_^2^ due to the matching of the shape of the stress field and the electric field (Figure 5a). Similarly, reflector layers can cause the loss of the stress field, thereby reducing the coupling. 

#### 2.3.2. Quality Factor

The Q of a resonator is defined by the device’s loss mechanism. Various loss mechanisms are possible, including acoustic losses, ohmic losses of the electrodes and viscoelastic losses [15]. Particularly, shear waves and laterally leaking waves can constitute a major loss mechanism for BAW resonators [16,17]. The electrode of the BAW resonator is usually not equipotential, thus eddy currents are produced, leading to I^2^R losses and decreased Q_s_ [18]. Because of the existence of the acoustic reflection layer, SMR-BAW may have more losses like material loss, incomplete suppression of energy loss which can flow through the reflection layer, and resistive losses from the interconnections. 

Since the calculation of Q is sensitive to spurious resonance and measurement noise, there are several approaches to estimate the Q of a resonator. Methods of 3-dB bandwidth and phase derivation are sensitive to the quality of the measured data [8], and can just characterize the Q-value at *f*_s_ and *f*_p_. The Bode method, or using the stored energy divide by the dissipated energy per cycle, can derive a Q that is not only at the two resonance frequencies, but also over a whole frequency range in the vicinity of those frequencies. A more specific analysis and discussion of Q have been given in [19]. 

### 2.4. Topology of Filter

**Figure 6:**
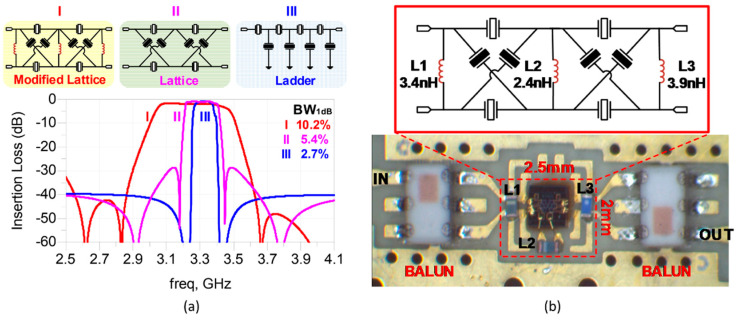
(**a**) Topologies of ladder type, lattice type, modified lattice with two auxiliary inductors, and their electrical responses [24]. (**b**) Board assembly of the filter with balun circuits [24]. Reproduced with permission from [24].

The BAW filters can be basically divided in ladder and lattice topologies. Generally, ladder-type filters present a steep rejection near the passband, but a poor out-of-band rejection. A. A. Shirakawa et al. presented a combined topology (ladder-lattice) that ally the advantages of both types with high selectivity and high isolation [20]. J. Verdu et al. modified the conventional ladder-type filter in order to improve the out-of-band rejection. By adding a series/shunt inductor, a new pair of transmission zeros will appear in the transmission response [21]. Then, they also developed a double-ladder configuration based on electrically coupled BAW resonators in order to obtain a dual-band transmission [22]. Since the bonding wires from the chip to the signal ports and ground should be considered when designing filters, Alexandre A et al. used the bonding wires with an inductive effect to improve the ladder-type filter return loss and isolation, overcoming the constraints given by the inductors association [23]. Qingrui Yang et al. [24] proposed a modified lattice network with two auxiliary inductors and the measured 3-dB bandwidth can reach 12.3% at 3.25 GHz (Figure 6).

## 3. Materials

### 3.1. AlN

At present, there are mainly three kinds of piezoelectric materials used in BAW resonators: AlN, PZT, and ZnO. For PZT, although it has a high *k*_t_^2^ and large dielectric constant, the inherent loss of PZT is too large, the speed of sound is too low, and the preparation process is difficult to keep the composition uniform. The *k*_t_^2^ of ZnO is also larger than that of AlN, but its longitudinal sound velocity is much smaller than that of AlN. Furthermore, the zinc element will reduce the carrier lifetime during processing and is not compatible with CMOS technology.

AlN is a hexagonal wurtzite structure with lattice constants *a* = 0.322 nm and *c* = 0.498 nm [25]. The maximum piezoelectric response (piezoelectric strain coefficient *d*_33_ is about 5.5 pC/N [26]) can be obtained in the longitudinal direction. Simultaneously, it has high stiffness *C*_33_ (3.67 × 10^11^ N/m^2^) [27], a relatively high *k*_t_^2^ (6.5–7%) [27], relatively high longitudinal sound velocity *v* (11354 m/s), and a low temperature coefficient (−25 ppm/°C or less) [28]. Other factors like the relative dielectric constant *ε*_r_ (about 10.2 [29]), the intrinsic Q of the material, and compatibility with CMOS processes are also consistent with the design requirement of BAW resonators and filters. Thus, piezoelectric AlN previously became the first choice for BAW resonators. 

AlN thin films can be deposited by magnetron sputtering, molecular beam epitaxy, pulsed laser deposition, and metal organic chemical vapor deposition. 

Among them, molecular beam epitaxy has a low deposition rate, high production cost, and poor process compatibility. Pulsed laser deposition method cannot be applied in large-area film deposition, and the thickness uniformity of the deposited film is poor. The metal organic chemical vapor deposition method is prone to cause large residual stress due to the high deposition temperature. At present, magnetron sputtering is the most used deposition method for preparing piezoelectric AlN films. It is generally performed at a lower temperature (typically 200–300 °C), and the maximum temperature is up to 400–500 °C during the reaction [30], which makes deposition processes compatible with CMOS technology and reduces deposition cycles. Besides, the method has the advantages of high efficiency, low cost, large deposition area [31], etc.

Rapid thermal annealing (RTA) after sputtering is another effective way to improve the quality of the AlN film. Vergara et al. found that after rapid annealing at 1300 °C, the intensity of the AlN (002) peak increased and the full-width half-maximum (FWHM) decreased [32]. Lin et al. used laser annealing to treat AlN. When AlN film was treated with a laser with a wavelength of 355 nm and a power of 0.025 W, the crystallinity of the film was significantly improved. The peak intensity of (002) was 58.7% higher than the untreated peak intensity [33]. However, high temperature and long treatment may have a negative impact [34], and the enhanced crystal quality of the films due to RTA hardly contributes to a significant improvement in *k*_t_^2^. In fact, results of RTA did not clearly describe the changes in thin film surface morphology, residual stress, and piezoelectric response, which makes RTA not used at the production level. 

**Figure 7:**
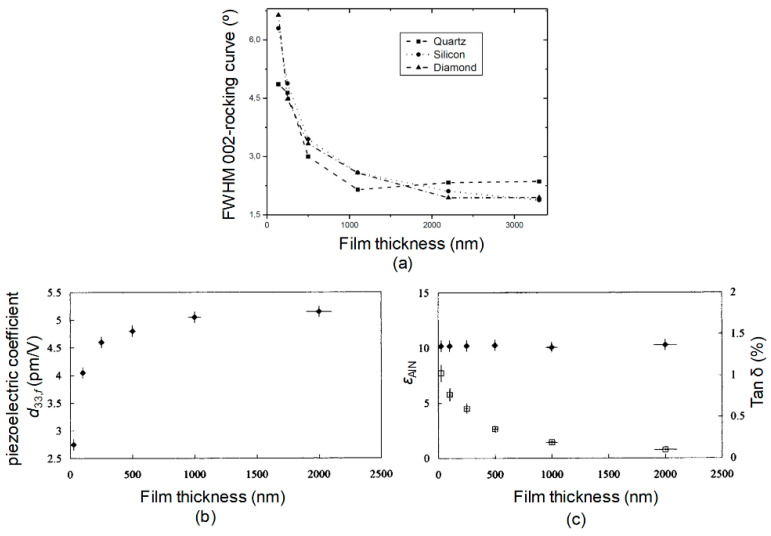
Effects of different thicknesses of AlN. (**a**) Rocking curve full-width half-maximum (FWHM) on different substrates, reproduced with permission from [35]. (**b**) Piezoelectric coefficient *d*_33_, reproduced with permission from [29]. (**c**) Permittivity (*ε*_AlN_ solid circles) and dielectric loss (Tan δ, open squares), reproduced with permission from [29]

In addition, the quality of AlN can be also affected by the thickness of films. Some studies have shown that as the thickness of AlN increases, the FWHM 002-rocking curve of AlN film decreases, *d*_33_ increases, and the dielectric loss decreases. When the thickness increases to 1 um, the change of thickness has little effect on the quality of AlN (Figure 7).

### 3.2. Single Crystal AlN

Compared with poly-crystalline AlN, single crystal AlN has quantifiable higher crystal quality, fewer defects, and more stable chemical properties. Generally, (002) X-ray diffraction rocking curve FWHM of single crystal AlN can be 0.02–1°, compared with the typical FWHM of 2–3° in poly-crystalline AlN. Improved crystal quality in the single crystal AlN has been proved to result in improvements in acoustic velocity and potentially improved piezoelectric coefficients [36]. Moreover, single crystal AlN-based BAWs are superior to the poly-crystalline AlN devices. Defects existing in poly-crystalline AlN will cause absorption or scattering of bulk acoustic waves and increase acoustic transmission loss.

However, there are still many problems that directly hinder the commercialization of single crystal AlN. For example, the area of single crystal AlN that we can obtain is usually small and normally based on a 2-inch [37,38] substrate; the thickness is small, usually only 100 to 200 nm [39]; and the film deposition is expensive, slow, and conditional [40].

**Figure 8:**
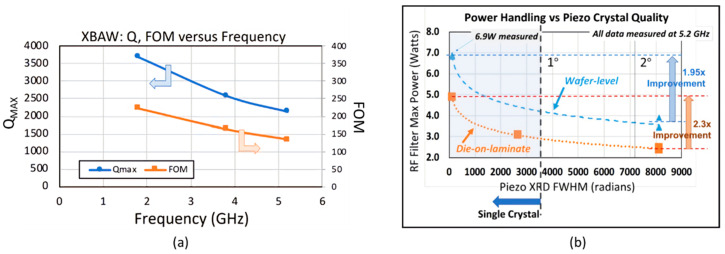
(**a**) Q-value and figure of merit (FOM) as functions of frequency when using XBAR^TM^ (single crystal AlN technology) [43]. (**b**) Measured power level capability of single crystal AlN and poly-crystalline AlN [43]. Reproduced with permission from [43].

In 2016, a group from Akoustis Technologies Inc. presented that single crystal AlN-based devices had more than double *k*_eff_^2^ than poly-crystalline AlN-based devices in the upper bound case, and the FOM is typically 30% higher, although the Q-value is slightly lower in single crystal devices [41]. In 2017, the same group used metal-organic chemical vapor deposition (MOCVD) to obtain single crystal epitaxial AlN film with 0.5 [42] and 0.6 [36] um thickness 4H silicon carbide (SiC) substrates with 150 mm diameter. Resonators showed *k*_eff_^2^ of 6.32% and 7.63%, and Q_max_ of 1523 and 1572, respectively. In 2018, a new high-power BAW device was created on a 6-inch Si substrate with high Q_max_ of 3685 and FOM of 222 working at 1.8 GHz (Figure 8a) [43]. Furthermore, experiments also confirmed that single crystal AlN devices had better power handling capabilities than AlN-based devices (Figure 8b).

Moreover, Shin et al. reported the growth of single crystal AlN by magnetron sputtering. They found that pre-depositing 5 nm thick Al on the substrate before single crystal AlN deposition is critical [44], which proved that the epitaxial growth of single crystal AlN on a Si substrate at room temperature by magnetron sputtering is possible. 

### 3.3. Doped AlN

Doping other elements into AlN is another effective method to improve the piezoelectric properties of AlN. Since Akiyama et al. found that the *d*_33_ of the piezoelectric film increased 400% after doping the Sc element [26], many efforts had been devoted to investigate the effect of doping elements on the piezoelectric properties of AlN films. 

The elements include Sc [26,27,45,46,47,48,49], Ti [50,51], Ta [25,52], V [52], Y [53], Mg [54], and Er [55]. The co-doping element includes Mg+Zr [56,57], Mg+Hf [57,58], Mg+Nb [59,60], and Mg+Ti [61]. Sc, Ta, Y, and Er increase the piezoelectric properties of the film within a certain doping concentration range. Ti, V, Mg, Zr, Hf, and Nb may deteriorate the piezoelectric properties of the film. Moreover, controlling the ratio of multi-element doping, such as Mg+Zr/Hf/Nb/Ti, can also greatly improve the piezoelectric properties of the film. 

**Figure 9:**
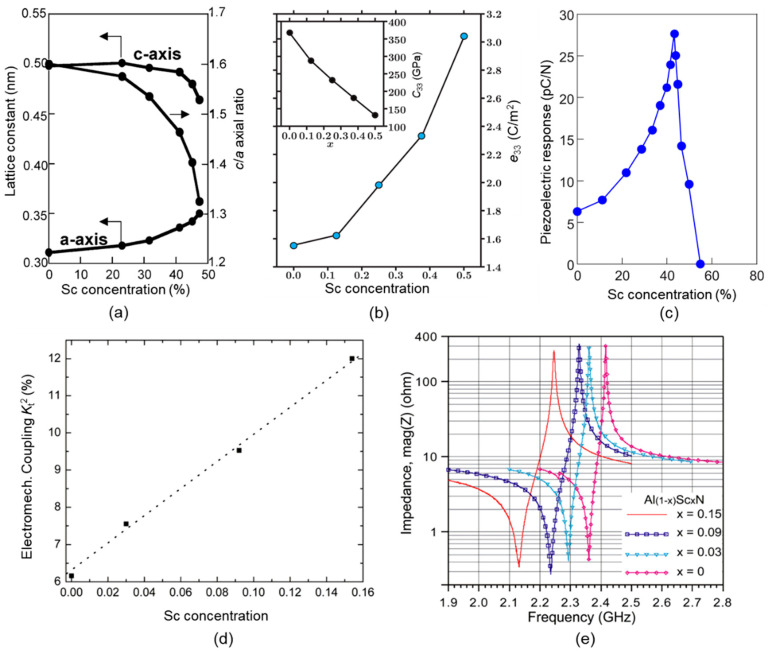
Effects of different Sc doping concentrations. (**a**) Lattice constant *a*, *c*, *c*/*a* value, reproduced with permission from [26]. (**b**) Piezoelectric constant *e*_33_ and stiffness coefficient *c*_33_, reproduced with permission from [46]. (**c**) Piezoelectric strain coefficient *d*_33_, reproduced with permission from [45]. (**d**) Electromechanical coupling coefficient *k*_t_^2^, reproduced with permission from [65]. (**e**) Measured FBAR impedance curve, reproduced with permission from [65].

The reason why doping certain elements into AlN can lead to an increase in the piezoelectric properties and *k*_t_^2^ of the piezoelectric film is that the lattice constant of the original unit cell will change when these elements are doped into AlN. For example, as Sc concentration increases, the lattice constant *a* will increase, and the lattice constant *c* will increase initially and decrease at a certain value of concentration. However, the overall value of *c*/*a* will decrease (Figure 9a) [26], which will cause the film to be more sensitive to pressure in the *c*-axis direction. 

Generally, these doping elements increase the piezoelectric coefficient *e*_33_, the relative dielectric constant *ε*_r_, and decrease the stiffness coefficient *c*_33_, thereby increasing the piezoelectric strain constant *d*_33_ of the material and the *k*_t_^2^ (*d*_33_ ≈ *e*_33_/*c*_33_ [46], *k*_t_^2^ = *e*_33_^2^/(*c*_33_ × *ε*_r_ + *e*_33_^2^) [27]) (see Figure 9). Unfortunately, these doping elements reduce Young’s modulus [48] of the film, the longitudinal sound velocity [62], and the temperature stability [62] of the piezoelectric film, and increase the dielectric loss [48].

On the other hand, although doping elements such as Sc can improve the piezoelectric properties of piezoelectric materials and increase the *k*_eff_^2^ of the BAW resonator, the Q-value of the resonator is generally reduced. The reason is that the doping element will increase the dielectric loss, thus deteriorating the quality of the piezoelectric material [63]. Zywizki et al. [64] studied the effect of different Sc doping concentrations on the quality of ScAlN films. They found that the FWHM of the (002) diffraction peak increased from 0.12° to 0.52° when the concentration of Sc increased from 0% to 36.8%. As the content of Sc continued to increase, the intensity of the peak gradually decreased until it disappeared. The introduction of the Sc element not only damages the orientation of the film but also reduces the uniformity of the film due to the segregation of elements. Therefore, it is important to control the doping concentration of Sc in ScAlN film for obtaining a good performance.

### 3.4. Electrode Material

In addition to the influence of piezoelectric materials, the choice of the electrode materials is also important. The ideal electrode material should have high acoustic impedance (high acoustic impedance difference with piezoelectric material is beneficial to improve the effective electromechanical coupling coefficient of the resonator), low resistivity (the lower the resistivity, the smaller the *R*_s_, which is beneficial to increase the Q-value of the resonator), and low density (higher density means higher mass loading, which is not conducive to the high frequency of the resonator). Moreover, since the piezoelectric material is directly deposited on the electrode material, the type and quality of the electrode directly affect the quality of the piezoelectric material. Good orientation, low surface roughness (reducing the scattering loss of sound waves [66]), and low lattice distortions with piezoelectric material are beneficial to improve the quality of the piezoelectric film and improve the performance of the resonator.

**Figure 10:**
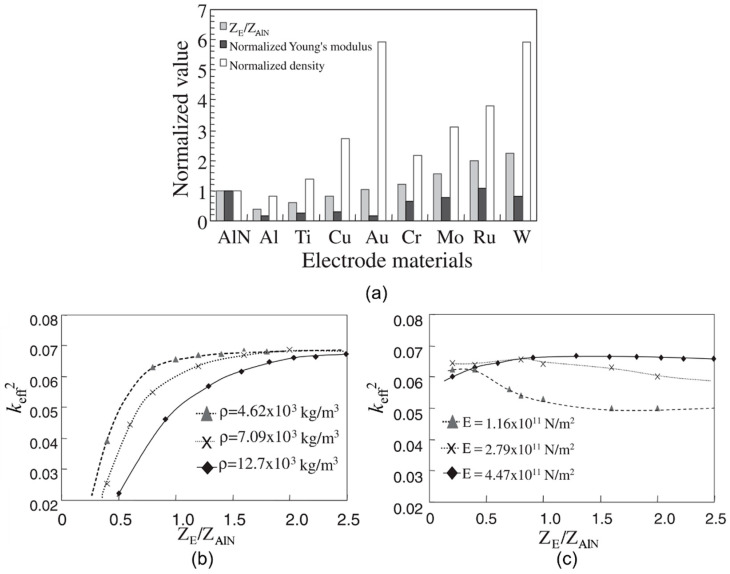
(**a**) Acoustic impedance, Young’s modulus, and density of electrode materials [71]. Relationship between *k*_eff_^2^ and normalized acoustic impedance with (**b**) *ρ* fixed and (**c**) *E* fixed [71]. Reproduced with permission from [71].

Many research works have studied the effects of various electrode materials, including Pt [67], Mo [66,68], W [69], Ru [70,71], Ir [72,73,74], Ta [75], Al/Au/Cu [76], CNT (carbon nanotube) [77], graphene [78,79], and ITO (indium tin oxide) [80]. Among them, Mo, W, Ru, and Ir have high Young’s modulus, but the density of W is relatively high. Al, Au, and Cu have lower resistivity, but their acoustic impedance is too low. Mo has a relatively moderate acoustic impedance, density, and resistivity, so it is the material most used as an electrode (Figure 10a). CNT and graphene are novel electrode materials, which have the advantages of low density and low electrical resistivity, but the preparation of electrodes is currently difficult. 

Masanori et al. further studied the effect of Young’s modulus *E* and density *ρ* on *k*_eff_^2^ ($Z=\sqrt{\rho E}$) [71]. It was found that when the *ρ* is fixed, *k*_eff_^2^ increases with the increase of the electrode *Z* (*E* increases) (Figure 10b), and when the *E* is fixed, the *k*_eff_^2^ changes slightly as the electrode *Z* increases (*ρ* increases) (Figure 10c). This indicates that *E* plays a major role in determining the increase of *k*_eff_^2^. It was also found that higher energy and smaller energy leakage are presented in the piezoelectric layer with higher *E*.

Lee et al. found that for the same material, a thin metal electrode reduces the Q-value of the resonator. The reason is that the thin film increases the resistivity of the material. Similarly, since the resistivity of W electrode is higher than that of Al electrode, even if the roughness of the W surface is low (small scattering loss), the increased ohmic loss will greatly reduce the Q-value of the resonator [66].

Akiyama et al. studied the effect of lattice matching on the growth quality of AlN. The results displayed that AlN and FCC (face-centered cubic) lattice structure of the electrode are matched very well, leading to a small FWHM of the (002) diffraction peak of AlN [76]. Table 1 summarizes the lattice structure of different metals. Yokoyama et al. studied the effect of the bottom electrode with different RMS values on the quality of the AlN film. It was found that a higher FWHM value of the (002) diffraction peak of the AlN film can be obtained with a higher root mean square (RMS) value [70]. In addition, Olivares et al. found that the surface of the bottom electrode can be cleaned by Ar+ bombardment before deposition of AlN, and the RMS value of the bottom electrode is reduced, thereby improving the quality of AlN [73]. Kamohara et al. found that a seed layer between the substrate and the bottom electrode greatly improves the quality of the bottom electrode, thus enhancing the quality of the AlN film [68,81].

## 4. Spurious Mode

For a BAW resonator, the spurious mode usually refers to the transverse vibration mode near the main resonance. The root source of the spurious mode is that a large number of vibration modes are coupled with the excitation electric field in a resonator of limited size [82,83]. Although these resonance peaks are relatively small, the existence of spurious modes still brings many hazards. Moreover, the spurious mode affects the *k*_eff_^2^ and Q of the resonator. Filters constructed by those resonators have worse insertion loss, smaller bandwidth, and increased ripple in the passband. In fact, the spurious mode can be reduced by increasing the area of the resonator, but this method is contrary to the miniaturization requirements of the resonator.

There are three main methods to suppress the spurious mode: apodization [84], adjusting the dispersion curve flatness of the resonator, and using the frame structure [85].

The apodization method adjusts the shape of the electrode so that the electrode does not have parallel sides, thus prolonging the path length of the acoustic wave propagation. On the other hand, the length of the transverse resonance path at the edge of the working area is unequal to prevent the formation of resonance peaks near the longitudinal wave frequency. In 2012, Park H et al. [86] studied the effects of circular, elliptical, rectangular, and trapezoidal electrodes on the electrical properties of resonators. Experimental results showed that the electrodes with asymmetric structure have less spurious modes and higher Q-values and *k*_eff_^2^ at the anti-resonance point than the electrodes with symmetric structure.

**Figure 11:**
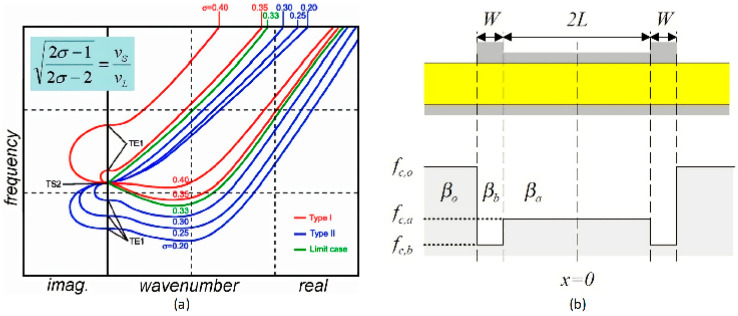
(**a**) Influence of the Poisson ratio onto the dispersion type of a BAW resonator. Poisson ration is used to correlate the longitudinal wave velocity and the shear wave velocity of the material, reproduced with permission from [85]. (**b**) The structure of suppression of the type I resonator and the cut-off frequency diagram, reproduced with permission from [14].

In general, a piezoelectric stack can only work with a certain dispersion type [87]. The method by adjusting the dispersion curve flatness is based on the dispersion curve analysis. By adjusting the flatness of the thickness extensional mode (TE1) branch of the dispersion curve, the spurious mode near the main resonance frequency is reduced. Fattinger et al. found that the dispersion type of resonator and the flatness of dispersion curve can be modified by varying the thickness of uppermost oxide in the SMR acoustic mirror [85]. The type of dispersion curve is dominated by the material properties, especially the Poisson’s ratio. When the Poisson’s ratio of the material is higher than 0.33, the resonator exhibits type I dispersion and when it is less than 0.33, the type II dispersion is achieved. For a type I dispersion curve, the cutoff frequency of the TE1 mode is greater than the cutoff frequency of the thickness shear mode (TS2) (*f*_TE1_ > *f*_TS2_), and the type II dispersion curve is just the opposite (*f*_TE1_ < *f*_TS2_) (Figure 11a). Particularly, a negative group velocity occurs on the TE1 branch when the type I dispersion curve is converted to the type II dispersion curve. The second part of the branch exhibits a positive group velocity at a high wavenumber, which may impair the electrical response of the device because the main resonant frequency corresponds to a relatively small number of waves. According to Table 2, since the Poisson’s ratio of AlN is 0.25, the AlN-based resonator generally exhibits a type II dispersion curve.

The apodization method and the flatting dispersion curve do not block the coupling of the transverse vibration and the excitation electric field, so there is still a problem about the leakage of lateral acoustic wave energy. The use of a frame structure based on the boundary conditions of the resonator directly avoids the generation of lateral acoustic waves. By analyzing and adjusting the dispersion curve of the resonator, the coupling can be blocked. At this time, the lateral displacement of the active region is uniform, called piston mode, while the displacement of the outer region is exponentially attenuated [83].

The boundary condition of the frame structure, the active area, and the outer area can be satisfied by choosing the frame structure with the appropriate width [88]. For Type I resonators, the spurious mode can be suppressed by adding a border structure to achieve the transverse wave vectors in the outer and border parts satisfying wavenumber *k*_o_ imaginary and *k*_b_ real, respectively [14]. It is necessary to ensure *f*_c,b_ < *f*_c,a_ < *f*_c,o_ (Figure 11b). At this time, the border is designed to superimpose a stacked layer higher than the thickness of the resonator at the edge of the resonator, called “raised frame-like”. On the contrary, Type II resonators need to exhibit *f*_c,b_ > *f*_c,a_ > *f*_c,o_, which means the frame structure needs to additionally contain a structure less than the thickness of the resonator. Therefore, the structure including a negative and a positive border is proposed, called “recessed frame-like” [88]. Both structures cause an exponentially decrease in the displacement outside the resonator, thus limiting the acoustic energy in the effective area.

In fact, the above three methods are applicable in both SMR and FBAR, and can be used together. But compared with FBAR, SMR has a greater degree of freedom in adjusting the dispersion curve due to the existence of acoustic mirrors, thus the spurious mode for SMR-BAW is easier to be suppressed.

**Figure 12:**
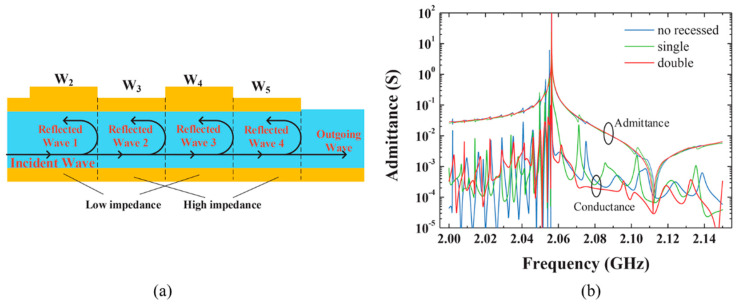
(**a**) BAW resonator with double raised frame [91]. (**b**) Admittance curves of structures with the double raised frame, the single raised frame, and without the recessed frame [91]. Reproduced with permission from [91].

For a single raised frame, energy leakage is influenced by the size of frame. If choosing an inappropriate frame size, some unwanted Lamb mode would be activated and increase acoustic wave power loss, which leads to a low Q-value [89,90]. In order to further reduce the mode-conversion and increase reflection coefficient at the frame region, Xinyi Li et al. [91] reported a double raised border of FBAR working in type Ⅱ piston mode. Through the double raised border frame, a high Q-value resonator can be obtained because of the high reflection of first-order symmetric (S1) Lamb mode for type Ⅱ dispersion (Figure 12). However, the *k*_eff_^2^ for a double raised border of FBAR will be reduced due to the enlargement of the non-active area. Similarly, a double-step frame with different height was proposed to reflect two Lamb modes with the largest energy at anti-resonant frequency [92]. The modified structure shows an improvement of Q_a_ of 82% compared to the no frame design. Furthermore, the double-step frame with thick metal contributes to enhancing the Q_r_.

**Figure 13:**
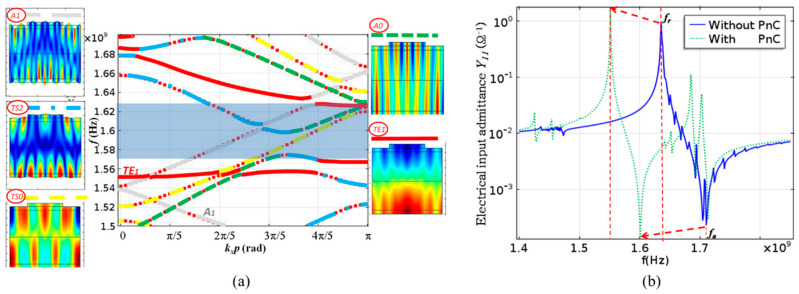
(**a**) Dispersion curve of acoustic waves in the FBAR; the formation of PnC directly bans the TE1 spurious mode generation at the antiresonance frequency [93]. (**b**) Comparison between the calculated input admittances of FBAR without PnC and with PnC. The resonant frequency decreases due to the mass-loading effect, and the curve becomes smooth as the Q-value increases [93]. Reproduced with permission from [93].

Since the frame structure strongly depends on the material and dispersion type, and will change the electrical properties of the BAW resonator, the phononic crystal structure (PnC) is proposed to suppress the spurious mode and improve the Q-value of the device. Phononic crystals use the theory of acoustic wave diffusion. This principle uses Bragg reflection to change the characteristics of the propagation medium by changing the periodicity and relative position of the scatterers. By adding the PnC structure, a bandgap can be formed on the dispersion curve, and the spurious mode in the bandgap is prohibited (Figure 13). Generally, the position of the bandgap can be adjusted be close to the main resonance frequency by controlling the structural size of the PnC [93].

## 5. Temperature Compensation

Since the sound velocity of material (such as AlN) becomes lower as the temperature rises, the operating frequency of a BAW resonator will become lower as the temperature increases. Generally, the method to improve the temperature coefficient of a BAW resonator is to insert a material that has a positive temperature coefficient frequency (TCF), such as silicon dioxide (SiO_2_), though it is known that deposition of SiO_2_ will decrease the *k*_eff_^2^ and Q-value. This approach achieves temperature compensation without any feedback control and external component.

**Figure 14:**
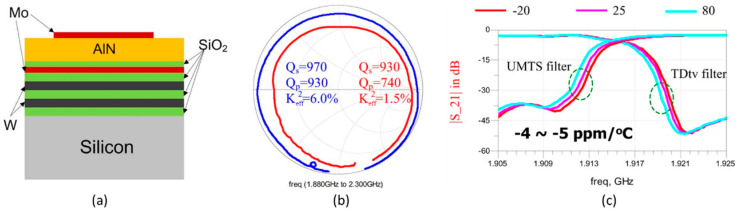
(**a**) Structure of the BAW resonator with a SiO_2_ temperature compensation layer [97]. (**b**) Measured impedances of the BAW resonator with a temperature coefficient frequency (TCF) layer and without a TCF layer [97]. (**c**) Measured temperature coefficient of frequency of Universal Mobile Telecommunications System (UMTS) band-pass filter and TDtv band-stop filter [97]. Reproduced with permission from [97].

There are many factors influencing the effectiveness of temperature compensation when using SiO_2_ as a compensated layer, such as the deposition position and the ratio between SiO_2_ and negative TCF layers [94,95,96]. In the case of the position of SiO_2_ layer, it is usually deposited on the topmost layer in a piezoelectric stack, which can protect resonator from moisture and contamination. However, this method will increase the mass loading sensitivity, making it difficult to trim the layer to obtain a desired frequency [96]. Hao Zhang et al. [97] fabricated a TC resonator by depositing SiO_2_ layer between the piezoelectric film and the bottom electrode. However, the electrical field falling on the SiO_2_ layer makes *k*_eff_^2^ of the resonator become smaller, and the measured Q-value also decreases. Band-pass and band-stop filters with excellent wireless interferences were designed to have a TCF of −4 ppm/ºC (Figure 14).

**Figure 15:**
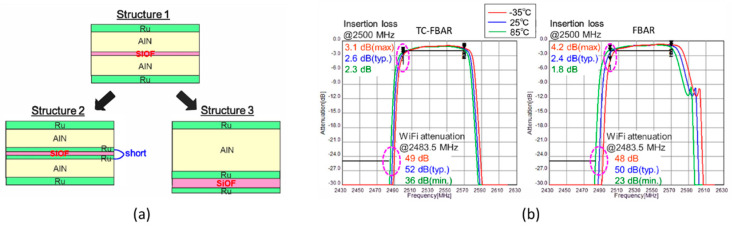
(**a**) Structure of the TC-FBAR with SiOF layer [99]. (**b**) Passband performance of TC-FBAR of structure 3 and conventional FBAR [99]. Reproduced with permission from [99].

On the other hand, in order to achieve a zero-drift frequency of the BAW resonator, a large amount of SiO_2_ (large ratio between the SiO_2_ and piezoelectric film) is usually used, which can deadly deteriorate the electrical character of BAW. Putting SiO_2_ at the high stress region of BAW is an excellent method, thus a minimum amount of SiO_2_ is used without large *k*_eff_^2^ reduction and Q-values loss [95]. Furthermore, a higher positive temperature compensation can be operated by using boron doped SiO_2_ or SiOF as the temperature compensation layer [98]. Tokihiro Nishihara et al. [99] studied three structures of TC-FBAR with SiOF layer (Figure 15a). Structure 2 and 3 eliminate the effect of parasitic capacitance, thus enhancing the *k*_eff_^2^ of TC-BAW, although the temperature compensation effect is slightly worse. The insertion loss at 2500 MHz under the temperature changing from 25 °C to −35 °C deteriorated from 2.6 dB to 3.1 dB and from 2.4 dB and 4.2 dB for the TC-FBAR and conventional FBAR, respectively (Figure 15b). These results indicated that the modified TC-BAW can effectively improve the temperature stability.

Compared with FBAR, SMR-BAW shows a huge potential in temperature compensation when using a positive material as the low impedance layer. According to the research [98], as the number of Bragg reflection layers increases, the TCF becomes smaller. In addition, increasing the thickness of the first SiO_2_ layer below the piezoelectric film (*d*_1_) can also decrease TCF. But when *d*_1_ increases from 0.25λ to 0.47λ, the *k*_eff_^2^ and Q-value decrease by about 15% and 13%, respectively. The reason is that the vibration energy stored in the first layer becomes larger, and more energy is leaked in the substrate. However, a novel design called an over-moded reflector, with SiO_2_ of 1/2 longitudinal acoustic wavelength thickness added to the first reflector layer in the SMR-BAW, was proposed recently. Though the thickness of the first layer of SiO_2_ increase, the increasing energy in SiO_2_ actually contributes to obtaining full temperature compensation and high Q-value [28].

M. DeMiguel et al. [100] also investigated the TCF of shear mode. They found that fully compensating the TCF of shear mode is challenging compared with longitudinal mode due to the bad response. A promising method is to make the reflector of SMR-BAW asymmetric or increase the number of the reflector layer.

One of the limitations of using positive layers is that zero TCF over a wide range cannot be achieved. Wei Pang et al. [101] raised an innovative method by integrating FBAR with an air-gap capacitor to solve this problem. The air-gap capacitor forms a cantilever and bends with the temperature changing, thus reducing the TCF of the FBAR by about 40 ppm/°C.

## 6. Frequency Tuning Ability

In the fabrication process of the BAW device, the resonant frequency is usually different from the target value due to the deviation in the process. Furthermore, the frequency offset caused by the temperature change, electromagnetic disturbances and aging also need to be considered. On the other hand, 5G frequency allocations for mobile phones vary widely among the world’s regions, so it is difficult for a filter with a single center frequency to cover all the bands. Considering the above two points, tunable BAW resonators and filters that can operate at different frequencies have received wide attentions in recent years.

At present, the BAW resonator tuning technology mainly includes: (1) changing the physical characteristics of the piezoelectric film by bias voltage; (2) tunable components such as passive elements; (3) a ferroelectric material with an electrostrictive effect; and (4) a micro-heater element [102] (by adjusting the temperature of the FBAR through a heater, but a relatively large electric power and time are needed to change the resonant frequency of the FBAR).

For the first method, the acoustic velocity depends on the stiffness that is a function of a direct current (DC) electric field. Thus, the resonant frequency of a BAW resonator varies as a function of applied DC voltage. However, this method can only supply a small amount of frequency tuning and requires a large DC bias [103].

Table 3 summarizes the impact of adding a series or parallel passive element to the BAW resonator. It can be clearly seen that capacitors and inductors only change one of the resonance frequencies of BAW resonator. In the case of adding a capacitance, the difference between *f*_s_ and *f*_p_ will be reduced, while the inductance is exactly the opposite [104,105].

However, placing discrete elements in the circuit would introduce parasitics and degrade the quality factor of the system. To avoid this disadvantage, Wanling Pan et al. [106] propose a structure with the piezoelectric plate suspended in air (or vacuum) above the bottom to form a MEMS various capacitor. This design not only reduces an assembly step during processing, but also promises a high-quality factor due to the elimination of interconnection loss. The continuous tuning range is limited to about 1.9 MHz at ~6.8 GHz with an actuation voltage of 9 V. Similarly, Wei Pang et al. [107] reported another structure of electrically tunable FBAR with an air-gap capacitor formed between the top electrode and piezoelectric film. Although integrating an air-gap capacitor in the FBAR can minimize the total size of FBAR, it may deteriorate the energy trapping in the lateral direction and make the FBAR vulnerable by the damaging effect of the surface micromachine.

Another way to realize frequency tuning is using tunable materials such as SrTiO_3_, BaTiO_3_, Ba*_x_*Sr_1-*x*_TiO_3_ (BST) to replace the piezoelectric layer of the devices. Those materials exhibit strong piezoelectric and/or electrostrictive properties. By applying a DC voltage, electromechanical coupling coefficient and sound speed of the device are changed, thereby achieving frequency shift. Particularly, because the BST thin film is the paraelectric phase, no resonance is observed at zero bias. However, with increased DC bias, the resonance develops and can be clearly tuned [108,109].

Table 4 summarizes the results of some existing studies on tunable BAW resonators using ferroelectric materials. Obviously, although those devices show some tuning abilities, the low Q-value inherent to ferroelectric materials limits the practical use.

**Figure 16:**
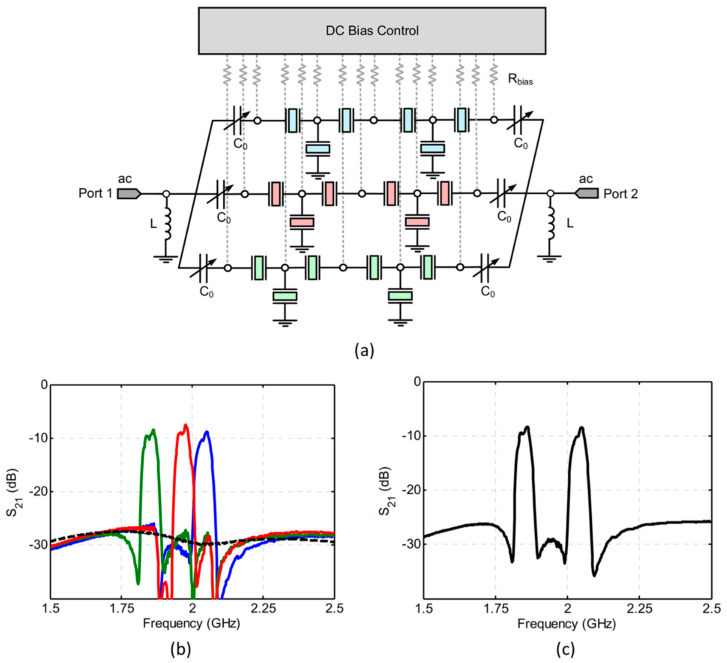
(**a**) Structure of the 2.5-stage BST switchable FBAR filter [116]. (**b**) Electrical response of the measured 2.5-stage switchable filter and the OFF-state response of the filter is provided in a dash line [116]. (**c**) Electric response of the filter when two switches are opened simultaneously [116]. Reproduced with permission from [116].

Moreover, the characteristics of tuning abilities contributes to the design of wide bandwidth and mutli-band filters, and the conventional circuit topologies limit the achievable bandwidth of the AlN BAW filter (typically 6%). As is described in Table 3, the introduction of inductance can lead to increased *k*_eff_^2^, thereby widening the effective bandwidth of filters. Seungku Lee et al. [116] designed and fabricated a switchable filter based on BST ferroelectric material using a 2.5-stage ladder-type configuration (Figure 16a). The filters were designed to have a 2.5% fractional bandwidth having center frequencies at 1.85, 1.96, and 2.04 GHz and provide an isolation of more than 27 dB (Figure 16b). For Carrier Aggregation (CA) applications, tuning on both channels simultaneously can achieve double/triple-band function (Figure 16c).

In fact, tuning frequency is complicated in acoustic resonators. Most of the above methods for tunable devices involve applying a variable electric field. The battery voltage of mobile phones is typically 2–3 V. In case a higher voltage is required for tuning, an intricate and space-consuming system is needed. Even if a suitable approach to tuning frequency can be found, there are many other conditions needed to be fulfilled for a tunable filter with any practical value.

## 7. Prospects

### 7.1. Lithium Niobate and Laterally-Excited Bulk-Wave Resonators (XBAR)

Lithium niobate (LN) appears to be a potential material with the form of crystalline films, due to the large value of its coupling coefficient for the fast shear mode.

**Figure 17:**
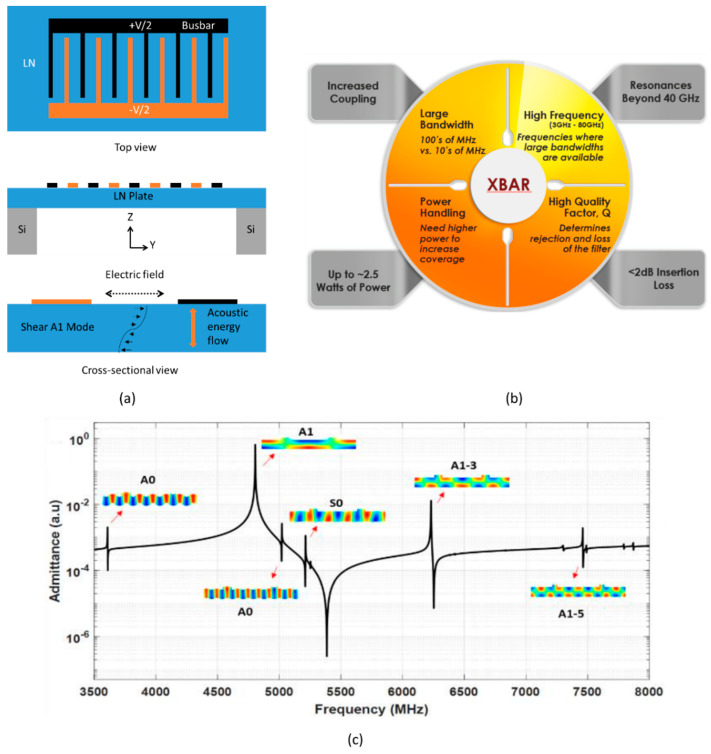
(**a**) The structure of XBAR with crystalline *z*-axis perpendicular to the platelet surface; shear wave resonance (A1 Mode) by applying lateral electrical field parallel to the crystalline *y*-axis [120]. (**b**) Key characteristics of the XBAR resonator addressing 5G requirements [123]. (**c**) Admittance curve from 2D periodic simulation indicating main XBAR mode and spurious mode [120]. Reproduced with permission from [120].

In fact, the traditional BAW technology cannot cover the wider bandwidths required due to the relatively small piezoelectric coupling. Therefore, a novel device named XBAR is proposed and receive continuous attention [117,118]. As shown in Figure 17, the resonator with interdigital transducers (IDT) on a suspended LN plate can excite standing bulk acoustic shear wave resonance by applying lateral electrical field parallel to the crystalline *y*-axis. The curve in the cross-section diagram represents the shear deformation in the LN layer, and small arrows indicate the direction and magnitude of atomic motion. The direction of acoustic energy flow of the primary shear acoustic mode is substantially orthogonal to the surface of the piezoelectric plate [119]. Experimental data showed that the main shear resonance is at 4.55 GHz, and the third harmonic is near 13 GHz. The relative bandwidth for shear bulk wave fundamental (A1) mode is about ~11% and the coupling coefficient *k*_eff_^2^ ≈ 25%, which absolutely satisfy the requirement for mobile phones at 4–6 GHz frequencies and even have huge potential at 10–25 GHz without harsh lithography conditions.

In addition to exciting vertical shear vibration A1 mode, there are also appearances of higher order harmonics of Lamb waves in the horizontal direction (A0, S0, A1–3, A1–5) (Figure 17c). Because the horizontal A1-3 harmonic is close to the right of anti-resonance, it may adversely affect the filter design. S. Yandrapalli et al. [120] found that changing the value of pitch and mass loading of metal electrodes can not only tune the frequency, but also adjust the relative position of A1-3 mode and anti-resonance. Furthermore, it is observed that there is no A1-3 mode at metallization of 25%, but magnitude of S0 and A0 mode increases.

A typical XBAR may have hundreds, possibly thousands, of parallel fingers in the IDT. The IDT of a XBAR differs substantially from the IDTs used in SAW resonators, which the pitch *p* of the IDT is typically 2 to 20 times the width *w* of the fingers. The width of the IDT fingers in a XBAR is not constrained to one-fourth of the acoustic wavelength at resonance [119]. However, there are still many problems to be solved for XBAR, such as poor mechanical reliability (large-size film hanging on the cavity), poor temperature coefficient of frequency, bad heat dissipation, and low power capacity. In addition, a cavity formed in the substrate can be replaced by the acoustic Bragg reflector, such as the multiple-layer structure in SMR-BAW. The acoustic Bragg reflector of SMR-XBAR [121] reflects the shear acoustic waves, thus keeping the acoustic energy predominantly confined to the piezoelectric plate. Inspired by SPH Loke’s work on two-dimensional (2D) Lamb wave resonators [122], varying the electrode dimensions and inter-electrode distancing to design two-dimensional XBARs may be an exciting idea.

### 7.2. Integration

In the 5G era, mobile phones need to work in multiband, which means that more RF front-end modules must be embedded in the same mobile phone space. As a result, the implementation of RF front-end systems is gradually transitioning from discrete devices to integrated chips [124]. Currently, module integration and monolithic integration are two feasible solutions.

“QM77031” is an integrated module launched by Qorvo that includes PAs, BAW filters and antenna switches. The main advantages of this product are that it can enhance performance, extend battery life, and reduce layout requirements. However, some manufacturers may feel that it has lost the flexibility of RF module design.

There are three potential monolithic integration concepts, including double-sided integration, single-sided vertical integration, and hybrid integration.

Double-sided integration mainly integrates different RF devices on the upper and lower sides of the same substrate though separate process; single-side vertical integration is a method of depositing thin films of different thicknesses on the same wafer to achieve resonators with different resonance frequencies; hybrid integration combines MEMS process and other standard process, which can integrate RF devices and IC components into a single module.

**Figure 18:**
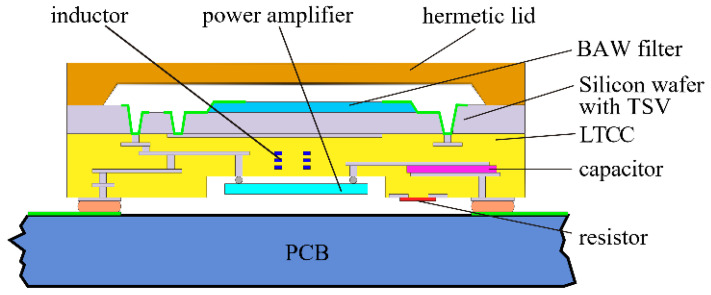
A hybrid integration module, including PA and BAW filter, with embedded passive components [125]. Reproduced with permission from [125].

In 2018, V. Chauhan et al. [125] proposed a valuable approach to the integrated design of PA plus BAW filter architecture in two different substrate technologies (epoxy/glass and SiCer). Using the LTCC and Si part of the SiCer substrate, a hybrid integration module embedded with passive components (resistor, inductor, capacitor) was proposed with short interconnections and reduced layout parasitics (Figure 18).

## 8. Conclusions

As a core component of the RF front-end, acoustic resonators/filters based on BAW technology will be widely used in base stations, automotive, electronics, radar, wireless communications. AlN-based BAW devices are still one of the best choices for Sub-6GHz due to their irreplaceable superiority in performance and manufacture.

In this review, the basic principle and key parameters of the BAW resonator have been described. The literature survey provides details on the different materials, design, and characteristics of the BAW resonator. High frequency, wide bandwidth, small size, and high-power capacity are the new challenges in 5G communication networks. It is difficult to complete a high-performance filter based on conventional BAW resonator technology. Doped AlN, single AlN, and LN are preferred materials to improve the inherent performance of the resonator. Research on resonator characteristics including spurious mode, temperature compensation, and tuning ability provide technical feasibility for high-performance, stable operation and application expansion of the filter. For mobile phone applications, integration of BAW devices could be an optional solution for miniaturization targets.

## Figures and Tables

**Table 1:** Crystal structure types of different metals (F: face-center cube; B: body-center cube; and H: close row hexagon) [55,57,60].

Metal	Au	Pt	Al	Ag	Cu	Ir	Mo	Cr	Nb	W	Fe	Ru	Ti	Co	Zn	Zr
Crystal structure	F	F	F	F	F	F	B	B	B	B	B	H	H	H	H	H

**Table 2:** The Poisson’s ratio of different materials. Data from [85].

Material	Al	W	MO	AlN	SiO_2_	Si_3_N_4_	Si	ZnO
σ	0.34	0.29	0.30	0.25	0.17	0.28	0.22	0.39

**Table 3:** Impact of adding capacitors and inductors to BAW resonators.

Series C	Parallel L	Parallel C	Series L
when C ↑	when L ↑	when C ↑	when L ↑
*f*_s_ → *f*_p_ *f*_p_ does not change	*f*_p_ ↑ *f*_s_ does not change	*f*_s_ ← *f*_p_ *f*_s_ does not change	*f*_s_ ↓ *f*_p_ does not change

**Table 4:** Performance of reported BAW resonators based on tunable materials.

Material	Freq (GHz)	Tuning Ability^1^ (%)	Max Bias (V)	Q^2^	Max *k*_eff_^2^ (%)	Reference
SrTiO_3_	5.87	*f*_r_:1	7.5	78(Q_s_)	2	[110]
BaTiO_3_	~4	*f_r_*:1.3 *f_a_*:4.0	10	30	6.2	[108]
Ba_0.25_Sr_0.75_TiO_3_	~4	*f_r_*:1.2 *f_a_*:0.8	10	120	0.5	[108]
Ba_0.25_Sr_0.75_TiO_3_	~5	*f_r_*:0.9	25	250(Q_s_) 350(Q_s-de_)	–	[111]
Ba_0.25_Sr_0.75_TiO_3_	5.46	*f_r_*:2	50	50(Q_s_) 140(Q_p_)	4.4	[112]
Ba_0.25_Sr_0.75_TiO_3_	5.76	*f_r_*:3.8	25	130(Q_s_)	7.1	[113]
Ba_0.25_Sr_0.75_TiO_3_	5.46	*f_r_*:1.9	50	260(Q_s-de_)	3.5	[114]
Ba_0.3_Sr_0.7_TiO_3_	2.85	*f_r_*:2.4 *f_a_*:0.6	615 kV/cm	200(Q_s_) 120(Q_p_)	4.4	[109]
Ba_0.5_Sr_0.5_TiO_3_	5.45	*f_r_*:2.4	50	250(Q_s-de_)	7.5	[114]
Ba_0.7_Sr_0.3_TiO_3_	5.44	*f_r_*:1.3	6	352.6(Q_s_) 746(Q_p_)	17.15	[115]

^1^ Tuning ability at resonance frequency *f_r_* and antiresonance frequency *f_a_*. ^2^ Q-factor at series resonance Q_s_, parallel resonance Q_p_ and series resonance after de-embedding Q_s-de_.
